# Growth-Mortality Relationships in Piñon Pine (*Pinus edulis)* during Severe Droughts of the Past Century: Shifting Processes in Space and Time

**DOI:** 10.1371/journal.pone.0092770

**Published:** 2014-05-02

**Authors:** Alison K. Macalady, Harald Bugmann

**Affiliations:** 1 University of Arizona, School of Geography and Development and Laboratory of Tree-Ring Research, Tucson, Arizona, United States of America; 2 Forest Ecology, Department of Environmental Systems Science, ETH Zürich, Zürich, Switzerland; University of Illinois at Chicago, United States of America

## Abstract

The processes leading to drought-associated tree mortality are poorly understood, particularly long-term predisposing factors, memory effects, and variability in mortality processes and thresholds in space and time. We use tree rings from four sites to investigate *Pinus edulis* mortality during two drought periods in the southwestern USA. We draw on recent sampling and archived collections to (1) analyze *P. edulis* growth patterns and mortality during the 1950s and 2000s droughts; (2) determine the influence of climate and competition on growth in trees that died and survived; and (3) derive regression models of growth-mortality risk and evaluate their performance across space and time. Recent growth was 53% higher in surviving vs. dying trees, with some sites exhibiting decades-long growth divergences associated with previous drought. Differential growth response to climate partly explained growth differences between live and dead trees, with responses wet/cool conditions most influencing eventual tree status. Competition constrained tree growth, and reduced trees’ ability to respond to favorable climate. The best predictors in growth-mortality models included long-term (15–30 year) average growth rate combined with a metric of growth variability and the number of abrupt growth increases over 15 and 10 years, respectively. The most parsimonious models had high discriminatory power (ROC>0.84) and correctly classified ∼70% of trees, suggesting that aspects of tree growth, especially over decades, can be powerful predictors of widespread drought-associated die-off. However, model discrimination varied across sites and drought events. Weaker growth-mortality relationships and higher growth at lower survival probabilities for some sites during the 2000s event suggest a shift in mortality processes from longer-term growth-related constraints to shorter-term processes, such as rapid metabolic decline even in vigorous trees due to acute drought stress, and/or increases in the attack rate of both chronically stressed and more vigorous trees by bark beetles.

## Introduction

Climate-related tree mortality has been documented in forests around the world and may be intensifying in some regions due to rising temperature and enhanced drought [1]–[6]. Forests play an important role in regulating the earth’s energy, carbon and water cycles [7], and increases in tree mortality rates or rapid collapses in forest cover could have major implications for ecosystems and ecosystem services [8]–[10]. Yet, our ability to predict future forest dynamics is limited by gaps in our understanding of tree death, and associated uncertainty about how represent tree mortality in vegetation models [6], [11]–[15].

The processes underlying drought-associated tree mortality are particularly unclear [16]–[20]. Leading hypotheses suggest that tree mortality may arise from two interrelated mechanisms: (1) carbon starvation, i.e., water stress causes trees to close their stomata, thus reducing photosynthesis and constraining the availability of carbon necessary for maintaining metabolic functions or fending off insects and pathogens; and (2) hydraulic failure, i.e., the collapse of the water-conducting system in the xylem [16], [21]. Much recent drought-mortality research has built on this framework in an experimental context to detect failure points within the linked hydraulic, photosynthetic and carbon transport systems of trees subject to drought, with the aim of clarifying mechanisms and identifying physiological and climatological thresholds beyond which death occurs (cf. [20], [22], [23]). However, our understanding of tree mortality remains inadequate for projecting the impacts of climate change on forests [6]. An important set of knowledge gaps relates to understanding *variability* in interacting drought-mortality processes and thresholds across landscapes and through time, and determining the time scales that are most important for understanding mortality risk, including the influence of previous events on the status and resistance of trees [22], [24], [25].

Tree ring studies offer an excellent way to complement more physiologically detailed but temporally and spatially limited experimental studies of drought-associated tree mortality. Radial stem growth is, at least in the short term, a low priority for a tree’s allocation of available carbon [26], [27]. Thus tree growth is often a sensitive indicator of changes in a tree’s carbon balance due to environmental or tree intrinsic factors. In the context of drought-associated mortality, for example, if trees close their stomata to reduce the risk of desiccation and hydraulic failure, carbon uptake and presumably radial growth are reduced. Hypothesized relationships between growth, a tree’s carbon budget, and mortality are reflected by mortality algorithms in many forest models, where recent (1–3 year) growth is a basis for determining the risk of death in a given time-step [11], [15]. Tree rings have been used to test and improve the empirical basis for such algorithms (cf. [28]–[32]). However, these studies have focused mostly on sporadic, individual-tree mortality, rather than on tree die-off associated with severe droughts. Furthermore, a paucity of long-term datasets has hindered the evaluation of the temporal and spatial generality of relationships between growth and the likelihood of death, although stable relationships are critical to the incorporation of growth-mortality algorithms in dynamic vegetation models [24], [33]. A number of studies have explored growth rates in trees that eventually died during drought to test hypotheses about the physiological mechanisms of mortality [34]–[43], but to our knowledge no studies have quantitatively assessed the importance of multiple growth variables and time scales for shaping mortality risk during drought-related die-off.

In the semi-arid conifer forests of western North America, prolonged drought and heat have interacted with bark beetles to produce mortality across millions of hectares during the last 20 years [5], [44]–[46]. In the piñon-juniper woodlands of the southwestern USA (SW), drought and the activity of bark beetles led to the widespread death of piñon pines (*Pinus edulis* Engelm. and *P. monophylla* Torr. & Frem) [5], [44], [47]. It has been suggested that several aspects of the 2000s drought and associated die-off were novel, and that unusually warm conditions caused elevated mortality rates that anticipate ‘global change’ conditions [5], [44], [48], [49]. However, tree mortality was also widespread in the SW during the 1950s [50], [51]. Although cooler than the 2000s drought across much of New Mexico [49], instrumental and tree-ring based records indicate that the 1950s drought was one of the most severe and protracted of the past 500 years [5]. Exceptional preservation of long-dead trees in these landscapes provides a unique opportunity to compare tree-ring growth and mortality patterns during the two mortality episodes, allowing not only for an evaluation of long- and short-term factors operating during widespread drought-associated die-off, but also an assessment of the generality of mortality processes and growth-mortality relationships during droughts with distinct climatic patterns.

In this study we draw on recent and archived tree-ring collections and stand structural measurements from sites spanning a latitudinal gradient in New Mexico, USA, to investigate drought-related mortality of piñon pine (*P. edulis* Engelm.). Specifically, we (1) analyze *P. edulis* growth patterns and mortality during the 1950s and 2000s droughts; (2) determine the influence of climate and competition on growth in trees that died vs. those that survived; and (3) derive regression models of growth-mortality risk and evaluate their performance across space and time.

## Materials and Methods

### Study Areas

We assessed *P. edulis* mortality close to lower treeline at four study areas that span gradients in climate and stand composition in New Mexico (Tables 1, 2; Fig. S1). Each area (TRP, BNM, WRK, SEV) is located on relatively gentle terrain at the middle-to-high end of the local elevation range of piñon, where the species co-occurs with one or more *Juniperus* species (*J. monosperma* and *J. scopulorum*). We refer to the study areas by acronym, and add 2000 or 1950 after each to distinguish between the sampling representing the two drought-mortality events.

**Table 1:** Location, characteristics and sample sizes for each study site.

Study Area	Location	Elevation (m)	Annual Precip. (mm)	Annual Temp. (C°)	Mortality event	Site Acronym	Live Target Trees	Dead Target Trees
Tres Piedras, NM	36.34**°**N 105.93**°**W	2100	312	8.7	2000s	TRP2000	29	30
White Rock, NM	35.81**°**N 106.24**°**W	1990	340	10.1	2000s	WRK2000	10	10
Bandelier Natl.Monument, NM	35.76**°**N 106.27**°**W	1940	340	10.1	2000s	BNM2000	0	28
					1950s	BNM1950	22	23
Sevilleta Natl. Wildlife Refuge, NM	34.34**°**N 106.55**°**W	2050	387	10.6	2000s	SEV2000	30	30
					1950s	SEV1950	27	26

**Table 2:** Mortality severity and stand characteristics for each study site.

Site	Piñonmortality (%)	JuniperMortality (%)	TreeDensity (stems/ha)	PiñonDensity (stems/ha)	TotalBasal Area(m^2^/ha)	PiñonBasal Area(m^2^/ha)
TRP2000	64.0 (4.1)	2.9 (1.7)	426.8 (36.8)	333.8 (36.9)	8.8 (0.9)	6.1 (0.7)
WRK2000	82.1 (7.5)	0.2 (0.2)	605.5 (78.1)	206.6 (34.4)	7.0 (1.1)	3.0 (0.6)
BNM2000	99.6 (0.4)	2.6 (1.0)	808.4 (71.5)	282.9 (41.0)	9.7 (1.1)	3.5 (0.7)
SEV2000	19.9 (3.2)	3.1 (1.0)	726.2 (49.0)	331.1 (31.3)	11.2 (0.8)	3.1 (0.3)
BNM1950	64.5 (7.5)	-	-	262.1 (26.5)	-	-
SEV1950	46.5 (8.5)	-	-	432.0 (61.0)	-	-

Standard errors for each measurement are indicated in parentheses. For 2000s sites, the mean and standard error were calculated based on 7.5m neighborhood plot data. For 1950s sites, the mean and standard error are from measurements at two separate 0.5-hectare (ha) plots. Tree density and basal area reflect pre-mortality conditions. No estimates of juniper mortality, total tree density or basal area were made for the 1950s sites due to lack of dendroecological data for juniper and lack of tree size reconstructions for all trees within each plot.

### Ethics Statement and Data Availability

This research was performed at the Sevilleta National Wildlife Refuge, Bandelier National Monument and the Carson National Forest. Necessary permissions were obtained from the National Park Service, National Fish and Wildlife Service and National Forest Service. All data are available upon request.

### Field Sampling and Laboratory Methods

#### 2000s mortality

In 2008–2010, we selected 10–30 recently dead (bark and fine branches remaining) “target” trees at each of the four study areas (Tables 1, 2). Living trees were selected as “control cases” to compare with each dead tree based on proximity, similarity in micro-topography, tree diameter and overall stature [29], [31]. Mortality processes can differ between adult and juvenile trees [52]; we selected trees greater than 9cm diameter at root collar (DRC) in order to focus our study on the mortality of mature trees [53]. Very few mature piñon trees were alive at BNM in 2010, thus 30 dead target trees were selected at this site for comparison to live and dead trees at the other sites.

Two increment cores were extracted from each living target tree at breast height (135 cm), and a cross-section was taken from each paired dead tree. In the laboratory, cores and cross-sections were prepared, crossdated and ring widths were measured using standard dendrochronological techniques [54]. We confirmed visual crossdating statistically using the computer program COFECHA [55]. In total, 167 (n = 98 dead and n = 69 live) target trees were sampled and successfully crossdated. One live target tree at TRP and two dead trees at BNM could not be crossdated, and were dropped from the study. For the characterization of stand structure and spatial patterns of mortality, we measured the diameter and noted the status (live or dead) and species of each tree (>1 cm DBH) within a 7.5 m radius plot centered on each live and dead target tree.

#### 1950s mortality

SEV and BNM are associated with a previous study for which long-dead trees were measured and sampled along with living neighbors (Allen, Betancourt and Swetnam, *unpublished data*). All living trees, snags and downed remnants within two 0.5 ha plots at BNM and SEV were measured, and each piñon tree was sampled for dendroecological purposes. These 0.5 ha plots fall within the larger sampling areas for each site described above. Each core and cross section was prepared and visually crossdated as above in order to determine inside and outside ring dates for each tree. To investigate growth-mortality relationships associated with the 1950s drought, we selected target trees from the archived specimens of this sampling campaign (all housed at the Laboratory of Tree-Ring Research, University of Arizona). We identified dead specimens with good preservation (e.g. where the last year of radial growth could reliably be determined) that were ≥9 cm DRC and had outer ring dates between 1940 and 1960. By many definitions, the 1950s drought actually stretched from the mid-1940s through the beginning of the 1960s [5], and so we allowed selection of trees with outer ring dates slightly preceding the first significant drought year in the 1940s (1946). Dead tree samples were measured and checked for dating errors as described above. We then identified and measured cores or cross-sections from trees ≥9 cm DRC that survived through the early 1960s. Growth increments from these samples were used to estimate tree diameter in the year 1960. Trees from this pool were matched as much as was possible to dead “case” trees based on the 1960-diameter estimate and 0.5 ha study plot. The pool of suitable survivor trees was limited by the fact that larger trees appear to have been preferentially killed during the 1950s drought at these sites. Ultimately we selected 25 dead and 26 surviving target trees for SEV1950, and 23 dead and 22 surviving trees for BNM1950, for a total of 96 trees, with the 1950s dataset containing slightly larger dead than surviving trees (Table 2). However, all trees met our size criteria (DRC ≥9 cm) and were estimated to be at least 65 years old by the 1950s drought.

### Target Tree Characteristics

Outside ring dates were recorded as the best-available approximation of the death date for each dead tree. Direct observations of dying trees at BNM2000 suggest that tree-ring estimates and actual death dates agree within a year or two (C. Allen, *personal communication*). Many insects and diseases are known to affect piñon pine, but *Ips confusus* LeConte – the piñon *Ips* bark beetle – has been associated with the most severe damage, and is known to attack both living and recently dead trees [56], [57]. We thus documented evidence of *Ips* attack for each dead target tree by noting whether an individual contained *Ips* beetle galleries on the sampled portion of the tree bole and/or whether the sample contained blue stain – a fungal pathogen introduced by bark beetles – in the sapwood [58].

### Woodland Structure and Spatial Patterns of Mortality

We calculated density and basal area of live and dead trees within neighborhood plots and used quasi-binomial regression to assess relationships between woodland structure and mortality severity (the percentage of recently dead trees) during the 2000s mortality event. We assessed fine-scale spatial patterning of recent mortality by testing for differences in stand composition and mortality-severity around live versus dead target trees.

It was not possible to make the same assessments of woodland structure prior to the 1950s mortality episode because of unknown tree locations within the 0.5 ha plots, but we made conservative estimates of piñon mortality severity using the dendrochronologically determined birth and death dates of the trees sampled on the 0.5 ha plots (cf. above).

The rings of juniper trees at our sites cannot be reliably crossdated due to many false and missing rings and lack of circuit uniformity, and therefore no assessments of juniper size, structure and mortality were made.

### Growth and Growth Indices

We calculated basal area increments (BAI), relative basal area increments (RelBAI) and ring width indices (RWI) from raw ring-widths (RW) for use in subsequent analyses. Basal area increments (cm^2^ yr^−1^) were calculated from ring widths (mm yr^−1^) for each tree radius using the inside-out method:
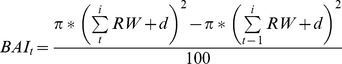
(1)where *d* is an estimate of the distance from the first measured ring to the pith [59], *i* is the first year of growth in the time series, and *t* is the current year of growth.

RelBAI (cm^2^ yr^−1^ yr^−1^) was calculated by dividing basal area increments for each year by the previous year’s total basal area [60]. We utilized the C-method to generate ring-width indices [61]. This method transforms ring widths by dividing individual series by a curve that reflects the biological expectation of constant annual basal area increment for each tree. The C-method thus standardizes individual ring width series to a common mean and variance, but unlike other standardization methods, it allows individual index series to retain low-frequency variability and trends due to, for example, injury, senescence, competition, and climatic influences. Measurements from multiple radii were averaged to generate single records of RW, BAI, RelBAI and RWI for each tree.

Although BAI is often considered to be a more biologically meaningful growth metric than raw ring-widths or ring-width indices [61], [29], [62], [39], we utilized RW for building models of growth-based mortality risk and assessing growth relationships to climate and competition, and RWI for the calculation of average growth chronologies. RWI was used in chronologies in order to minimize the influence of particular trees with high mean growth and variance, and to highlight changes in the *trajectory* versus the average growth rate in dead versus surviving trees. We utilized RW in quantifying growth-mortality relationships because, unlike RWI, it retains gross differences in growth rates between live and dead trees, and contrary to expectation, we found less pronounced size-related trends in piñon RW compared to BAI and RelBAI (Fig. S2). Although data from our neighborhood plots indicate that tree size was likely a predisposing factor for mortality at our 2000s sites (Fig. S3), our study design is not well-suited to quantify the combined influence of tree size and growth on mortality risk, because (1) the 1950s dataset is slightly biased towards larger dead trees due to preservation issues; and (2) average sampled tree size (and age) was not stratified between study sites (Table 2). Thus even though tree size and age matched relatively well between living and dead trees and target trees shared similar dominant or co-dominant status, we sought to choose a growth metric that is least sensitive to tree size in order to make a conservative estimation of the growth-mortality relationships. For the sake of comparison, we also analyzed basic growth differences between live and dead trees using all metrics, and generated growth-mortality models using BAI, RelBAI and RWI as outlined below. These models had slightly different predictor variables and performance, but did not lead to different conclusions and thus are not shown.

We generated four types of indices from growth time series to develop a pool of predictor variables of mortality risk: average growth, growth variability, growth trend and the frequency of abrupt growth changes. Average growth [16], [26] was calculated as the mean of annual growth measurements over *k* = 3, 5, 7, 10, 15…50 years. Growth variance has been documented as a factor that influences predisposition to mortality in semi-arid woodlands [35], [36]. We chose mean sensitivity, a statistic of year-to-year growth variability that reflects both the variance and the first-order autocorrelation of the time series [63], because compared to standard deviation (Table 2) or first-order autocorrelation (not shown) it differed more strongly between living and dead trees. Mean sensitivity was calculated from growth time series according to Eq. 2 in Biondi and Quedan [64], where *k*  =  the length of the tree-ring series *t* = 1,2,…,k  =  year in the tree-ring series:
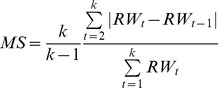
(2)Growth trend was calculated as the slope of the linear regression of growth fitted across all years within different time intervals [29]. Both mean sensitivity and growth trend were calculated over *k* = 5, 10, 15…50 years.

Several studies have noted a preponderance of abrupt growth declines in trees predisposed to die [31], [34], and we also noted differences in the frequency of growth releases in our dataset. We defined abrupt growth changes as 50% reductions or increases in growth averaged over a 10 year period as compared to average growth over the previous 10 years. We counted the number of such changes over *k* = 5, 10, 15…50 years.

For the calculation of all predictor variables, live tree growth was truncated at the last year of the corresponding dead tree pair.

### Mortality Modeling

We used linear mixed effects logistic regression to relate growth indices to tree status (live or dead) [65], [66]. We followed the general procedure of Das et al. [31], modified for mixed effects modeling, to identify the most parsimonious model structure. We designated study site/period as a random effect. For the fixed effects, we: (1) generated models with only one covariate using a temporal range of growth indices from each growth category (average growth, growth trend, mean sensitivity, and abrupt growth changes). We did not include indices after 35 years because multiple trees with shorter crossdated growth records dropped out of the predictor pool after this point, making it difficult to compare models; (2) used Akaike’s Information Criterion (AIC) to compare the support for each model [67]; and (3) created a suite of models with multiple fixed effect predictors using the variables from the three single-variable models with the lowest AIC score in each growth category and/or with differences in AIC scores below 2 [67]. Independent variables were transformed if Wald tests indicated non-linear relationships [68]. Random effects were dropped from each model if AIC scores and likelihood ratio tests on nested models indicated that a simpler model structure was more parsimonious [69]. The three models with the lowest AIC scores overall are presented along with the best-ranked single variable models for comparison.

### Model Diagnostics, Validation and Interpretation

We computed a variety of diagnostic and validation statistics to aid in the interpretation of best-ranked logistic models. Correct classification rates were calculated from confusion matrices generated by a bootstrapped internal validation routine (1000 iterations) in which models were fit repeatedly with a random sub-sample containing 60% of the data and validated on the remaining 40% [29]. Trees were classified as living if their survival probability was above an empirically determined threshold [70]. We also externally validated best-ranked models on the dataset from BNM2000, which contained only dead trees, did not match the case-control study design, and thus could not be used in model building. However we expected that the best models would correctly classify the majority of dead trees at BNM2000 if mortality processes and thresholds were similar here compared with other sites. The Area Under the Receiver Operating Characteristic curve (ROC) is a threshold-independent measure of model discrimination, where a value of 0.5 suggests no discrimination and values above 0.8 suggest excellent discrimination between live and dead trees [71]. Odds ratios were calculated from regression coefficients to assess changes in relative mortality risk associated with changes in growth. Odds ratios indicate a *change* in the likelihood of mortality given a meaningful change in the predictor variables. For example, an odds ratio of 2.0 associated with a 0.1 mm increase in average growth can be interpreted as a doubling of the likelihood of survival with each 0.1 mm growth increase, all else being equal.

### Effects of Climate and Competition on Tree Growth and Mortality

We fit a separate set of linear mixed-effect models to make *post-hoc* assessments of how two factors - climate and competition - influenced growth in trees destined to die and survive drought-mortality events [41]. For target trees at TRP, WRK and SEV, we modeled ring width as a function of cool season precipitation (previous September through May) (PPT*_cool_*), early summer (May–July) average vapor pressure deficit (VPD_MJJ_), a continuous index of competitive pressure (CI), and a categorical variable representing tree status (Live or Dead). The seasonal climate variables were chosen based on initial comparisons between mean-value chronologies and PRISM climate model output (4 km resolution) [72] (Text S1). PRISM data were chosen instead of weather station data because PRISM data better explained the variability in tree growth chronologies (not shown).

Indices of competitive pressure (CI) were derived from the neighborhood plot data (cf. above). A distance- and size-weighted index, calculated using only conspecific (e.g. piñon) neighbors was used for modeling growth, as it yielded the strongest and most consistent correlations with recent growth:
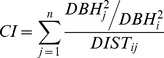
(3)where *j* = 1,…,n are competitor trees, *i* is the target tree, DBH is tree diameter and DIST is the distance between target and competitor trees [29]. For analyses of the 1950s sites, competition was not included as a covariate because neighborhood data were not available. Growth, climate and competition variables were converted to z-scores specific to site and the modeled period.

Model fitting reflected our goals of assessing the effects of climate and competition on tree growth, and testing whether trees destined to die and survive responded differently to climate and competition in the years leading up to mortality. First, through exploratory analyses, we noted that the relationship between ring width and climate is sometimes slightly curvilinear, and models without higher-order climate terms contained highly skewed residuals. Thus, following [69] we started with a ‘beyond-optimal’ model that included as fixed effects both linear and quadratic climate variables along with tree status, competition, and site. Because of the potential complexity of interactions in a model with a high number of predictors, we restricted the ‘beyond-optimal’ model to 2 and 3-way interactions that represented interpretable biological processes [69]. We did not include interactions between climate variables, as our initial analyses indicated that they were small. Interactions between quadratic climate terms and other predictors were not included, either. Tree ID was added as a random effect to account for non-independence of growth within individual trees. More complicated random effects structures (for example, allowing different growth trends over years for different trees, or nesting Tree ID within site) were rejected, as they did not improve the full model. Residual autocorrelation of growth between years and heterogeneity of variance in residuals were accounted for by adding model correlation structures and variance weights [69], [65], [41].

We used an iterative, AIC-based backwards selection to sequentially drop terms from the ‘beyond-optimal’ model [69]. We developed a final model structure using growth data that started in 1960 and 1910 for the 2000s and 1950s datasets, respectively. We retained this structure for models of growth over different time periods to allow for the straightforward comparison of coefficients. Residuals were inspected to ensure that assumptions about residual independence, heterogeneity and normality were adequately met. Final models were fit using the restricted maximum likelihood criteria. Growth data from BNM2000 were not included in model fitting, as there were no living trees.

Unless otherwise noted, all statistical tests were performed in R v3.0.1 [73]. We used the *dplR* library v1.5.6 for BAI and RWI calculations and dendrochronology statistics [74]. Generalized linear mixed modeling was performed using functions from the *lme4* library v0.99999911-6 [75]. Linear mixed models of tree growth were fit using the library *nlme* v3.1-110 [65].

## Results

### Spatial and Temporal Mortality Patterns

The severity of piñon mortality during the recent (2000s) drought ranged from 20% to 99% across our study sites, and was least severe at SEV2000 (Table 2). Piñon mortality associated with drought in the 1940s and 1950s was also severe at both SEV1950 and BNM1950 (45%–65%), with slightly higher mortality at BNM1950 (Table 2). These measurements are based on 7.5 m neighborhood plot data around each target tree (2000s drought) or dead and live trees in 0.5 ha plots (1950s drought), and thus reflect mortality severity at our study sites only. However, for the 2000s drought the patterns in our data conform to other published studies with more extensive sampling, in which the 2000s mortality was found to be greater in northern versus south-central New Mexico [76].

**Figure 1:**
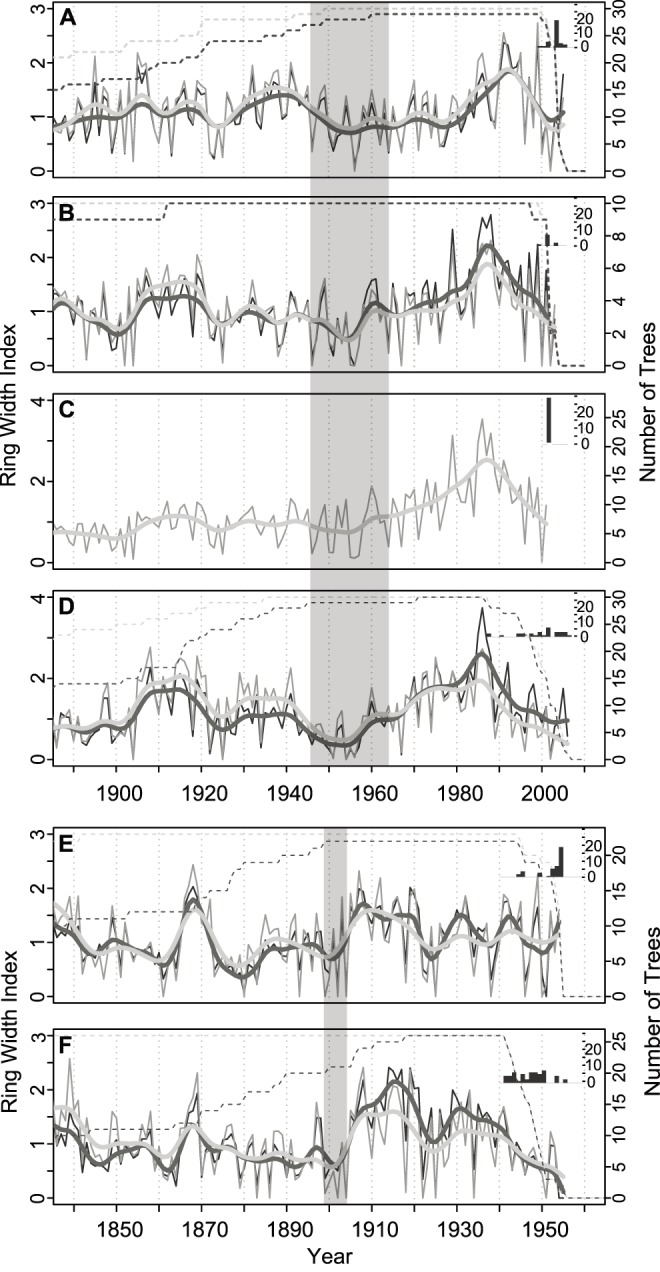
Growth chronologies and death dates from piñon target trees. Live (black) and dead (grey) tree ring-width index chronologies for TRP2000 (A), WRK2000 (B), BNM2000 (C), SEV2000 (D), BNM50 (E), and SEV50 (F). Tukey’s biweight robust mean was used to calculate chronology values from individual index series. A smoothing spline (df = 40) (thicker lines) is overlain on the annual mean value chronologies (thinner lines). A horizontal dashed line indicates the number of trees contributing to chronologies in each year. Bar plots of outside ring dates for dead trees at each site are shown in the small panels within each larger time series panel. The transparent grey boxes show SW drought events (as defined in [5]) preceding the 2000s (A–D) and 1950s (E–F) mortality events. The period 1945–1964 was the sixth strongest drought event since 1000 A.D., and the period 1899–1904 was the seventeenth strongest.

Outside ring dates correspond well with periods of decadal drought, but mortality was more or less synchronous depending on site and mortality episode (Fig. 1). Fine-scale spatial patterning of mortality at the 2000s study sites varied along the latitudinal gradient, with a non-significant positive relationship between tree density and mortality severity at TRP2000 grading into a weakly significant negative relationship between density and mortality severity at SEV2000 (Fig. S4). Finer-scale clumping of dead piñon was also characteristic of mortality at the northern sites in the 2000s (TRP2000 and WRK2000), with more piñon trees and more dead piñon trees around dead versus living target trees (not significant at the 0.05 level at WRK2000). No such clustering existed at SEV2000 (Table S1). Drought-associated mortality was generally concentrated in medium-sized to larger trees at the 2000s sites, based on size-class distributions of living and dead piñon trees measured in neighborhood plots (Fig. S3).

### Evidence of Insect Attack

Evidence on sampled cross-sections points to the almost ubiquitous presence of *Ips* beetles during both mortality events. Seventy-nine percent of dead trees in the 2000s dataset contained *Ips* galleries on sampled bole cross-sections, and 94% had sapwood colored by blue stain fungus. At least one type of evidence was present on 97% of dead samples. All samples with no evidence of successful bark beetle attack were from the SEV2000 site, where 10% of dead trees had no indication of activity. In our 1950s dataset, 67% of samples exhibited *Ips* galleries on sampled cross-sections, and 100% contained blue stain fungus.

### Differences in Growth between Dead and Surviving Trees

Averaged across all sites, trees that survived drought had higher recent growth rates (53% higher 3-yr average RW, p<0.001) and lower average mean sensitivity (18% lower 20-yr mean sensitivity, p<0.001) than dead trees. Average recent growth and mean sensitivity exhibited the same direction of difference between live and dead trees at all sites where both live and dead trees were sampled, although differences were only significant at SEV2000 and the 1950s sites (Table 3; Figs. S5–S6). Significant differences in growth trends and abrupt growth changes were present between live and dead trees at some sites, but they were less consistent in magnitude and direction (Table 3; Figs. S7–S8). Differences in average growth and mean sensitivity extended back many decades at some sites, yet they were generally minor and insignificant earlier in the life of trees (Figs. S5–S6). At some sites with stronger growth differences between live and dead trees, a divergence in growth occurred after previous severe and/or protracted drought intervals (e.g., after the 1950s for 2000s sites, and after a drought at the turn of the century for the 1950s sites; cf. Fig. 1).

**Table 3:** Average size and growth characteristics of live and dead target trees.

	TRP2000		BNM2000		WRK2000		SEV2000		BNM1950		SEV1950	
	Live	Dead	Live	Dead	Live	Dead	Live	Dead	Live	Dead	Live	Dead
Diameter (cm dbh)	15.9	16.1	-	13.6	17.4	17.2	11	11.9	**8.8**	**12.1**	**7.7**	**10.7**
***Recent average growth***												
3yr RW (mm/yr)	0.328	0.234	-	0.476	0.357	0.248	**0.412**	**0.157**	**0.439**	**0.239**	**0.358**	**0.131**
3yr RWI	0.824	0.765	-	1.186	1.337	1.036	**1.329**	**0.686**	1.146	1.102	**1.013**	**0.662**
3yr BAI (cm^2^/yr)	1.771	1.284	-	1.948	2.005	1.431	**1.389**	**0.602**	1.162	0.927	**0.830**	**0.466**
3yr RelBAI (cm^2^/yr/yr)	0.009	0.006	-	0.016	0.010	0.006	**0.017**	**0.006**	**0.017**	**0.007**	**0.020**	**0.005**
***Long-term average growth***												
20yr RW (mm/yr)	0.609	0.514	-	0.859	0.460	0.348	**0.578**	**0.319**	**0.442**	**0.236**	**0.521**	**0.220**
20yr RWI	1.488	1.614	-	1.954	1.668	1.406	**1.805**	**1.341**	1.200	1.087	**1.366**	**1.077**
20yr BAI (cm^2^/yr)	2.840	2.290	-	2.958	2.286	1.915	**1.738**	**1.184**	1.015	0.886	1.103	0.784
20yr RelBAI (cm^2^/yr/yr)	0.020	0.017	-	0.037	0.013	0.009	**0.027**	**0.012**	**0.020**	**0.008**	**0.031**	**0.008**
***Growth variability***												
20yr Mean Sensitivity (RW)	0.509	0.563	-	0.501	0.578	0.591	**0.419**	**0.536**	**0.521**	**0.729**	**0.478**	**0.694**
20yr Mean Sensitivity (RWI)	0.515	0.569	-	0.508	0.581	0.596	**0.423**	**0.544**	**0.528**	**0.732**	**0.477**	**0.693**
20yr Mean Sensitivity (BAI)	0.523	0.577	-	0.515	0.588	0.598	**0.426**	**0.540**	**0.538**	**0.733**	**0.475**	**0.692**
20yr Standard Deviation (RW)	0.310	0.282	-	0.432	0.229	0.178	**0.295**	**0.205**	**0.232**	**0.158**	**0.261**	**0.139**
20yr Standard Deviation (RWI)	0.783	0.898	-	1.014	0.828	0.724	0.915	0.742	0.651	0.739	0.667	0.669
20yr Standard Deviation (BAI)	1.401	1.252	-	1.489	1.132	0.947	0.944	0.879	0.589	0.596	0.568	0.482
***Growth trend***												
20yr Trend (RW)	−0.02	−0.02	-	−0.04	−0.01	−0.01	−0.02	−0.02	−**0.01**	**0.00**	−0.02	−0.01
20yr Trend (RWI)	−0.03	−0.06	-	−0.07	−0.04	−0.04	−0.06	−0.08	−0.01	0.00	−0.04	−0.03
20yr Trend (BAI)	−0.04	−0.06	-	−0.07	−0.04	−0.05	−0.05	−0.07	0.00	0.00	−0.03	−0.03
***Abrupt growth changes***												
20yr Increases (RW)	6.41	5.43	-	3.68	4.70	3.90	3.70	2.27	1.00	0.91	0.70	1.35
20yr Increases (RWI)	7.41	6.10	-	5.18	5.30	5.00	4.60	2.80	1.41	1.65	1.52	1.54
20yr Increases (BAI)	8.34	6.70	-	8.18	5.70	5.20	**7.43**	**3.43**	3.50	1.87	**4.15**	**2.12**

Significant differences between live and dead trees within sites are indicated by boldface type (p<0.05, Student’s t-test). No live trees were sampled for this study at BNM2000.

### Growth-based Models of Mortality Risk

**Figure 2:**
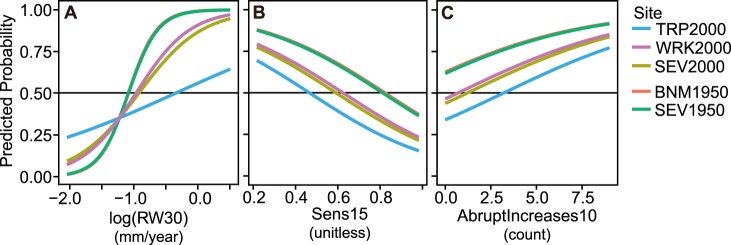
Predicted probabilities of mortality associated with the best-ranked logistic regression model. The figure shows predicted survival probabilities associated with the best-ranked growth-mortality model in Table 4. Values for mean sensitivity over 15 years (Sens15) and the count of abrupt growth increases over 10 years (AbruptIncreases10) are held at their mean for the dataset in (A); AbruptIncreases10 and log(RW30) (average 30-year growth rate) are held at their mean in (B); log(RW30) and Sens15 are held at their mean in (C).

The best logistic growth-mortality models were highly significant and resulted in good discrimination between live and dead trees, with ROC scores above 0.80 and correct classification rates slightly above 70% (Table 4). Models containing multiple and longer-term growth variables featured substantially lower AIC scores and higher discrimination statistics than models that included only recent growth as a predictor. The best-ranked model included an average growth variable, a measure of year-to-year growth variability, and a term characterizing abrupt growth increases (Table 4). The relative survival probability associated with each predictor varied depending on site (Fig. 2). The direction of growth-mortality relationships was consistent across sites included in the model-building dataset. However, the strength of growth-mortality relationships was weaker overall for the 2000s sites (and at TRP2000 in particular), and the threshold of mortality varied. Specifically, trees at the 1950s sites exhibited lower growth and higher growth variability than trees at the 2000s sites while still featuring survival probabilities above the empirically determined mortality threshold in our model (0.497) (Fig. 2).

**Table 4:** Best-ranked growth-mortality models.

*Model Form*		*Model Diagnostics and Validation*						
Fixed Effects	Random Effects	Δ AIC	ROC	ROC*_boot_*	DeadTreesCCR	LiveTreesCCR	AllTreesCCR	kappa
log(RW30) + Sens15+ AbruptIncrease10	1+ log(RW30) | site	0.00	0.847	0.844	72.6%	72.7%	72.6%	0.453
log(RW30) + Sens20+ AbruptIncrease10	1+ log(RW30) | site	2.20	0.846	0.848	71.5%	71.4%	71.5%	0.429
log(RW25) + Sens15+ AbruptIncrease10	1+ log(RW25) | site	2.80	0.842	0.838	73.5%	71.5%	72.5%	0.450
**Best 2-variable**								
log(RW30) + Sens15	1+ log(RW30) | site	5.90	0.834	0.826	71.6%	73.4%	72.5%	0.450
**Best 1-variable**								
*Average growth*								
log(RW20)	1+ log(RW20) | site	12.90	0.816	0.812	71.4%	69.4%	70.4%	0.408
*Growth sensitivity*								
Sens15	-	31.20	0.746	0.736	67.3%	67.5%	67.4%	0.348
*Growth trend*								
Trend15	-	71.90	0.584	0.580	48.9%	57.5%	53.2%	0.064
*Abrupt growth changes*								
AbruptIncrease10	-	73.00	0.512	0.517	53.4%	43.3%	48.4%	−0.032
**Recent Average Growth**								
log(RW3)	-	20.90	0.773	0.782	72.4%	72.9%	72.6%	0.453

Variables include average growth (RW), mean sensitivity (Sens), growth trend (Trend), and abrupt growth changes (AbruptIncreases), with the number of years over which variables were averaged indicated after variable type. The best single-variable models in different growth categories, along with a model containing recent average growth as the only predictor variable (log(RW3)), are shown for comparison. **Δ**AIC is the difference in AIC between the best-ranked model and the model shown in each table row, with smaller values indicating more parsimonious model fit. ROC is a threshold independent measure of model discrimination, where 0.5 suggests no discrimination and values above 0.8 suggest excellent discrimination. Correct classification rates (CCR) are based on a bootstrapped internal validation with 1000 iterations in which 60% of the data was used for model fitting and 40% was used for model validation. Trees were classified as living if model output was greater than the empirically defined threshold [70]. ROC*_boot_* is an average of the ROC statistics generated in the model-fitting portion of the bootstrapping routine. The kappa statistic measures the proportional improvement of the model classification over a random assignment of tree status [99], and was also estimated by taking an average of kappa statistics generated in the bootstrapping routine.

Odds ratios for the best-ranked model indicate that, averaged across sites, a 0.1 mm increase in RW averaged over 30 years increases the relative odds of survival by 1.32, all else being equal. Likewise, a 0.1 increase in mean sensitivity over a 15-year period decreases survival odds by 0.72, and one additional growth increase leads to an increase in survival probability by a factor of 1.2. Model coefficients and 95% confidence intervals generated in the bootstrapping routine indicates that all terms with the exception of the random slope term for average growth are significant at the 95% level (Table S2). We nonetheless retained the random slope term, given consistent improvements in AIC and significant increases in the log-likelihood compared to models without this term [69].

Although the best-ranked models produced satisfactory correct classification rates when evaluated internally, they performed poorly when externally validated on the BNM2000 dataset, significantly under-predicting mortality (only 12% of dead trees were classified correctly) (Table 5). Best-ranked models also performed unevenly when internally validated at the site level (Table 5). Site-specific correct classification rates indicate good model performance at the 1950s sites and SEV2000, but correct classification rates were not much better at TRP2000 and WRK2000 than if trees were classified by chance. Fitting growth-mortality models only on data from those individual sites did not dramatically improve this outcome (not shown).

**Table 5:** Site-specific correct classification rates for best-ranked growth-mortality models.

	*Correct Classification Rates*		
Site	Dead Trees	Live Trees	All Trees
TRP2000	60.0–63.3%	62.1%	61.0–62.7%
WRK2000	60.0%	60.0–70.0%	60.0–65.5%
BNM2000	12.0%	-	12.0%
SEV2000	**73.3%**	**80.0–83.3%**	**76.7–78.3%**
BNM1950	**82.6%**	**95.5%**	**88.8%**
SEV1950	**90.1%**	**77.3%**	**84.1%**

Each column shows the range of correct classification rates for the three best-ranked general mortality models. Bold typeface highlights correct classification rates that are consistently above 70%.

### Effects of Climate and Competition on Growth and Mortality

**Figure 3:**
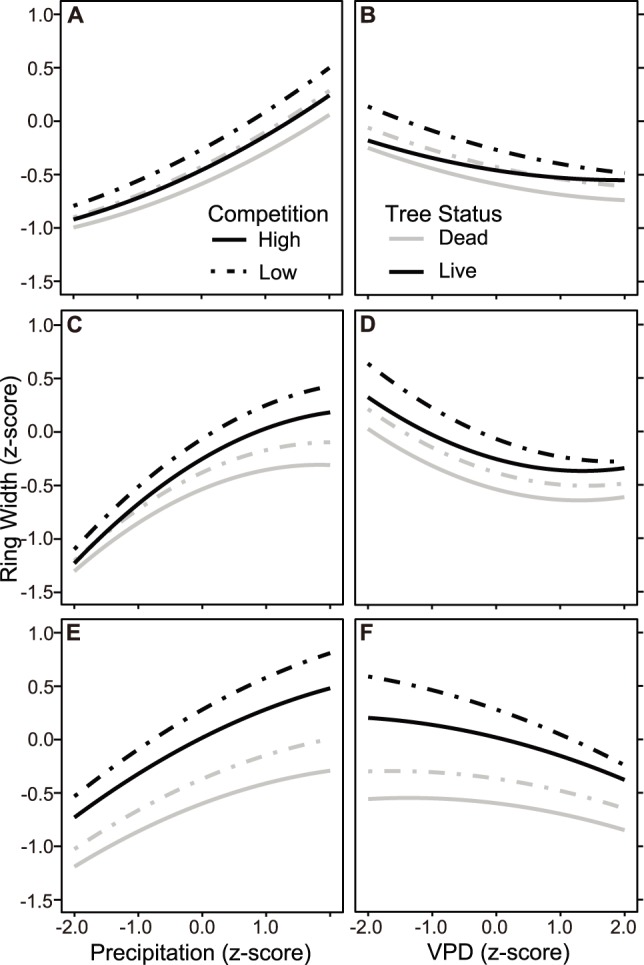
The predicted effects of precipitation (PPT*_cool_*), vapor pressure deficit (VPD*_MJJ_*) and competition (CI) on growth in surviving and dying trees from 2000s sites. The relationships reflect the model shown in Table 6. High and low competition levels are set to 75th and 25th percentiles of CI, respectively, with the predicted effects shown separately for TRP2000 (A, B), WRK2000 (C, D) and SEV2000 (E, F).

**Figure 4:**
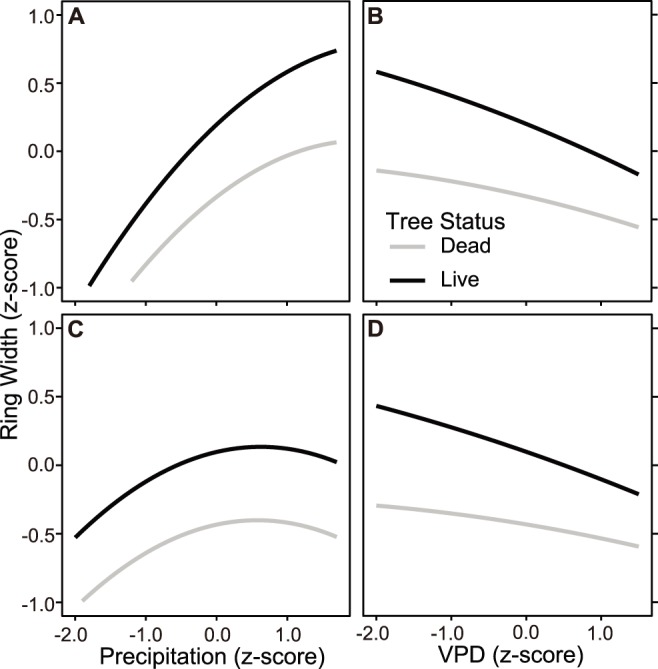
The predicted effects of precipitation (PPT*_cool_*) and vapor pressure deficit (VPD*_MJJ_*) on growth in surviving and dying trees from 1950s sites. The relationships reflect the model shown in Table 7. Predicted effects are shown separately for BNM50 (A, B) and SEV1950 (C, D).

We used linear mixed-effects models to make *post-hoc* assessments of the influence of climatic and competitive factors on radial growth in trees that survived and eventually died. These models confirm that growth over the decades prior to drought-mortality events was different depending on eventual tree status (live/dead), significantly and positively related to precipitation (PPT*_cool_*), and negatively related to growing-season VPD (VPD*_MJJ_*), though the effect of tree status and the slope of the growth response to climate varied across sites (Tables 6–7; Figs. 3–4, S9–S10). A tree’s growth was related to eventual tree status more strongly and consistently in 1950s and SEV2000 trees, confirming a generally stronger growth-mortality signal when compared to WRK2000 and TRP2000.

**Table 6:** The modeled relationship between tree growth, status, competition and climate in trees from 2000s sites.

	*TRP2000*				*WRK2000*				*SEV2000*			
Model Terms	β	SE	t-value	P-value	β	SE	t-value	P-value	β	SE	t-value	P-value
Intercept	−**0.51**	**0.06**	−**7.84**	**<0.001**	−**0.47**	**0.15**	−**3.19**	**0.002**	−**0.49**	**0.08**	−**5.95**	**<0.001**
PPT*_Cool_*	**0.28**	**0.01**	**19.09**	**<0.001**	**0.26**	**0.03**	**9.20**	**<0.001**	**0.24**	**0.02**	**14.86**	**<0.001**
(PPT*_Cool_)^2^*	**0.03**	**0.01**	**4.05**	**<0.001**	−**0.07**	**0.01**	−**6.95**	**<0.001**	−**0.04**	**0.01**	−**5.01**	**<0.001**
VPD*_MJJ_*	−**0.13**	**0.01**	−**8.89**	**<0.001**	−**0.17**	**0.03**	−**5.49**	**<0.001**	−**0.08**	**0.01**	−**5.51**	**<0.001**
(VPD*_MJJ_)^2^*	**0.02**	**0.01**	**4.29**	**<0.001**	**0.06**	**0.01**	**4.58**	**<0.001**	−**0.03**	**0.01**	−**3.34**	**0.001**
CI	−**0.11**	**0.04**	−**2.50**	**0.014**	−0.10	0.11	−0.91	0.362	−**0.15**	**0.06**	−**2.47**	**0.015**
Live Status	0.14	0.09	1.55	0.124	0.30	0.24	1.24	0.217	**0.63**	**0.14**	**4.59**	**<0.001**
CI:Live Status	−0.02	0.06	−0.38	0.707	−0.02	0.06	−0.38	0.706	−0.02	0.06	−0.38	0.707
PPT*_Cool_*:CI	−**0.02**	**0.01**	−**3.01**	**0.003**	−0.02	0.03	−0.73	0.466	−0.02	0.01	−1.73	0.084
PPT*_Cool_*:Live Status	0.03	0.02	1.38	0.169	**0.10**	**0.05**	**2.00**	**0.045**	**0.08**	**0.03**	**2.65**	**0.008**
VPD*_MJJ_*:CI	0.01	0.01	1.26	0.207	0.01	0.01	1.26	0.207	0.01	0.01	1.26	0.207
VPD*_MJJ_*:Live Status	0.01	0.02	0.27	0.790	−0.03	0.05	−0.60	0.549	−**0.10**	**0.03**	−**3.73**	**<0.001**
VPD*_MJJ_*:CI:Live Status	**0.03**	**0.01**	**2.40**	**0.017**	**0.03**	**0.01**	**2.40**	**0.017**	**0.03**	**0.01**	**2.40**	**0.017**

Summary of the linear mixed-effects model with the formula RW∼PPT*_Cool_* + (PPT*_Cool_*)^2^+ VPD*_MJJ_* + (VPD*_MJJ_*)^2^+ CI + Tree Status + Site + CI:Tree Status + PPT*_Cool_*:CI + PPT*_Cool_*:Tree Status + VPD*_MJJ_*:CI + VPD*_MJJ_*:Tree Status + PPT*_Cool_*:Site + (PPT*_Cool_*)^2^:Site + VPD*_MJJ_*:Site + (VPD*_MJJ_*)^2^:Site + CI:Site + Tree Status:Site + PPT*_Cool_*:CI:Site + PPT*_Cool_*:Tree Status:Site + VPD*_MJJ_*:Tree Status:Site + VPD*_MJJ_*:CI:Tree Status, random  =  (∼1 | TreeID). A correlation term and variance weights were also included in the model in order to account for residual autocorrelation of growth between years and variance heterogeneity of residuals by TreeID and across fitted values [65]. Growth was modeled from 1980, with the end of the modeled period varying depending on the outer growth year of the dead tree in each pair. Model parameters with estimates of (p<0.05) are in boldface type. The reference level for Tree Status is Dead. Contrasts were applied to calculate coefficients and significances associated with each site.

**Table 7:** The modeled relationship between tree growth, status, and climate in trees from 1950s sites.

	*BNM50*				*SEV50*			
Model Terms	β	SE	t-value	P-value	β	SE	t-value	P-value
(Intercept)	−**0.34**	**0.06**	−**5.47**	**<0.001**	−**0.43**	**0.05**	−**9.10**	**<0.001**
PPT*_Cool_*	**0.40**	**0.02**	**20.31**	**<0.001**	**0.11**	**0.01**	**8.33**	**<0.001**
(PPT*_Cool_)^2^*	−**0.10**	**0.01**	−**13.48**	**<0.001**	−**0.10**	**0.01**	−**13.48**	**<0.001**
VPD*_MJJ_*	−**0.13**	**0.01**	−**8.79**	**<0.001**	−**0.09**	**0.01**	−**6.10**	**<0.001**
(VPD*_MJJ_)^2^*	−0.02	0.01	−1.24	0.2149	−0.01	0.01	−0.99	0.320
Live Status	**0.53**	**0.07**	**7.41**	**<0.001**	**0.53**	**0.07**	**7.41**	**<0.001**
PPT*_Cool_*:Live Status	**0.08**	**0.03**	**2.53**	**0.0116**	0.01	0.02	0.42	0.6774
VPD*_MJJ_*:Live Status	−**0.10**	**0.03**	−**3.65**	**<0.001**	−**0.10**	**0.02**	−**4.45**	**<0.001**

Summary of the linear mixed-effects model with the formula RW∼PPT*_Cool_* + (PPT*_Cool_*)^2^+ VPD*_MJJ_* + (VPD*_MJJ_*)^2^+ Tree Status + Site + PPT*_Cool_*:Tree Status + VPD*_MJJ_*:Tree Status + PPT*_Cool_*:Site + (PPT*_Cool_*)^2^:Site + (VPD*_MJJ_*)^2^:Site + Tree Status:Site + PPT*_cool_*:Tree Status:Site + VPD*_MJJ_*:Tree Status:Site, random  =  (∼1 | TreeID). A correlation term and variance weights were also included in the model in order to account for residual autocorrelation of growth between years and variance heterogeneity of residuals by TreeID and across fitted values [65]. Growth was modeled from 1930, with the end of the modeled period varying depending on the outer growth year of the dead tree in each pair. Model parameters with estimates of (p<0.05) are in boldface type. The reference level for Tree Status is Dead. Contrasts were applied to calculate coefficients and significances associated with each site.

Interaction terms between tree status, PPT*_cool_* and VPD*_MJJ_* provide evidence of a differential response to climate amongst surviving and dying trees (Tables 6–7). Survivors from both the 2000s and 1950s exhibited a generally greater response (steeper slope) to precipitation than dying trees, driven by an enhanced growth response during wet years (Tables 6–7; Figs. 3A,C,E, 4A,C). Interactions between tree status and VPD*_MJJ_* indicate that surviving trees also usually had a greater growth response to VPD*_MJJ_*, driven by enhanced growth during years when atmospheric vapor demand was low (Tables 6–7; Figs. 3B,D,F, 4B,D). However, the interactions between tree status and climate were weaker and less consistent at TRP2000 (Table 6; Figs. 3B, S9).

Competition played a complex yet significant role in modulating tree growth. Growth was negatively affected by the presence of conspecific neighbors, and competition also reduced the ability of trees to grow well during years with abundant PPT*_cool_*, although this effect was weaker at WRK2000 and SEV2000 than at TRP2000 (Table 6; Figs. 3A,C,E, S9). Competition also modulated the response of trees to VPD*_MJJ_*, but this effect was contingent on tree status, with live trees with low CI best able to exploit years with low atmospheric demand, and the response of dying trees to VPD*_MJJ_* not significantly affected by competition (Table 6; Figs. 3B,D,F, S9).

The magnitude and significance of the growth predictors shifted slightly through time, but the direction of effects remained generally consistent (Figs. S9, S10).

## Discussion

### Long-term Factors Predispose Trees to Die during Drought Events

Radial tree growth serves as a proxy for tree status in the years preceding death, integrating the effects of drought, injuries, and tree structural characteristics on the carbon dynamics that can lead to mortality during prolonged and severe droughts. We observed consistently lower average growth in trees that died versus trees that survived, and significantly greater year-to-year growth variability. The latter may be related to carbon reserves and thus the relative capacity of trees to buffer themselves against inter-annual swings in resource availability [39], [77], although we cannot prove a physiological link via our data. Lower average growth and higher mean sensitivity among dying piñon are consistent with previous studies in other semi-arid forests [35], [36], [42], and with the hypothesis that piñon death during the droughts of the 1950s and 2000s was related at least in part to constraints on carbon uptake and/or storage, leading to lower growth and, ultimately, the inability to meet metabolic requirements or repel attacking insects [15], [16], [39].

More surprising than the observed growth differences *per se* is the fact that at some sites these differences extend over multiple decades, and yet were not present early in the growth records of trees (Figs. 1, S5–S8). This is consistent with the decline-disease theory of tree death [52], [78], and suggests that long-term processes or the contingent effects individuals’ response to previous events are underlying at least a significant portion of the mortality during two large, seemingly sudden die-off events. We propose two non-exclusive processes that are consistent with our observations.

First, growth in surviving and dying trees appears to diverge most strongly after previous record-setting decadal droughts, at least at some sites (Fig. 1). Some trees may have reacted to these droughts by shedding leaf or root mass [21], [79], [80], other physiological adjustments [21], and/or they may have sustained injuries such as loss of xylem conductance [25], [81], [82]. As a result of such responses, some trees became more vulnerable to mortality during subsequent drought [78]. This is consistent with findings on much shorter time scales in *P. sylvestris*, where drought and herbivory-associated reductions in leaf area reduced carbon uptake and reserves and influenced mortality risk during a subsequent drought year [21], [83]. It is also consistent with the inclusion of the abrupt growth increase term in our best growth-mortality model (Table 4), which reflects how *recovery* from periods of lower growth (and presumably higher stress) is important to mortality risk beyond the influence of average growth rate alone.

Second, the mortality of neighbors during previous decadal drought may have freed survivors from competition, allowing some to recover faster and/or boost their productivity. The lack of significant differences between spatial neighborhoods around dead and surviving trees at SEV2000 and WRK2000 (Table S1) – sites that exhibited the strongest growth divergences – along with weak relationships between mortality severity and tree density or basal area in neighborhood plots (Fig. S4), suggests that overall tree density was not the most important factor driving mortality risk among the trees in our study. However, the negative influence of conspecific neighbors on piñon growth, and the negative interaction between tree climate response and competition suggests that, although the effect may be complex, competition contributes to growth trajectories, and by extension, likely influences drought-mortality risk (Fig. 3).

The decades-long divergences in growth between dying and surviving trees that we observed are in agreement with those documented in a few other long-term studies [34], [36]–[38], and suggest that understanding tree recovery after drought may be critical to understanding the full impacts of drought-mortality events and anticipating future tree mortality. For example, would the 2000s die-off have been worse if the drought had occurred a decade sooner, when fewer trees had sufficiently recovered from the 1950s drought? Was the severity of mortality during the 2000s drought contingent upon the character and timing of the 1950s drought and the climate in the following years? Past divergences in growth also suggest that there is now a new pool of vulnerable trees that were injured but not killed during the early 2000s drought [25], further highlighting that potential changes in the *frequency* of drought may dictate the *severity* of future die-off events, along with changes in drought intensity and duration.

These results and interpretations are consistent with the view of extreme ecological events put forth by Gutchick and BassiriRad [84], in which the consequences of such events are hypothesized to become most evident during a long recovery period. Among our trees, structural adjustments or injuries caused by previous severe drought (as hypothesized above) might have had a genetic component [84], and the associated fitness costs in terms of lost growth potential proved fatal, even if decades later. Thus, drought-mortality processes may be more fully understood if, in addition to quantifying the instantaneous effects of climate on tree carbon and hydraulic dynamics, we expand the consideration of the controls on prolonged recovery from severe drought events, which may determine how individuals respond to environmental variability years to decades later [21], [84].

### Dying Trees Exhibit a Differential Response to Climate

The significantly different growth rates of dying vs. surviving trees leading up to the 2000s and 1950s droughts are partly due to differential responses to precipitation and VPD (Tables 6–7; Figs. 3–4, Figs. S9–S10). McDowell et al. [39] found that growth of *P. ponderosa* that died during the 2000s drought was more responsive to a drought index than growth of surviving trees, with dying trees growing less during the drier years leading up to mortality. They suggest that this is consistent with trees predisposed to die by low leaf-level gas exchange and carbon uptake driven by chronic water stress. However, Ogle et al. [35] found that growth in mature drought-killed piñon in Arizona was less responsive to drought variability than in survivors, and Millar et al. found that limber pine (*P. flexilus*) [42] and whitebark pine (*P. albicaulis*) [43] that died were less responsive to decreasing water deficit than survivors, at least at high temperatures.

We found that trees predisposed to die exhibited higher mean sensitivity (e.g. higher year-to-year growth variability, Table 2). Previous researchers have suggested that high mean sensitivity reflects greater limitation by inter-annual swings in climate or other environmental variables (e.g. [77] and see above). Although dying trees grew less than survivors during hotter, drier years (Figs. 3–4), our models suggest that growth in dying piñon was generally *less* responsive to the overall range of PPT*_cool_* than in survivors (Tables 6–7; Figs. 3–4, S9–S10). The response of dying trees to VPD*_MJJ_* was also generally less pronounced than among survivors, though this effect was reduced or negligible at TRP2000. Importantly, these differences were driven by enhanced growth of surviving trees during wet or cool, rather than dry or hot years (Figs. 3–4). This suggests that, in addition tree response to drought stress *per se,* the ability to maximize photosynthesis and growth during years with abundant water supply and low VPD may be an important aspect of tree survival during subsequent severe or prolonged drought. Recent evidence generated by precision dating of ^14^C in carbon within and respired by trees points to the utilization of years-to-decades-old stored carbohydrates for functions such as dormant-season metabolism, defense and repair [85]–[88]. Trees that survived may have been able to store excess carbon from enhanced photosynthesis during wet periods, and to use this carbon for defense and metabolism during subsequent drought periods when growth and photosynthesis were severely constrained [89].

### Competition Reduces Tree Growth and Modulates Trees’ Ability to Respond to Favorable Climate

High stand density has been found to increase the likelihood of mortality via competition in many forests [29], [52], [78]. However, recent studies of drought-associated mortality patterns in southwestern woodlands have documented variable relationships between tree density (or basal area) and the severity of mortality within a stand or site [53], [76], [90]–[92]. The proportion of dead trees in the neighborhood plots at our study sites was also inconsistently related to tree density or basal area (Fig. S3). However, growth of piñon trees across sites was negatively influenced by the presence of conspecific neighbors, with CI also reducing the ability of trees to take advantage of wet and/or cool conditions over the decades before drought (Table 6; Figs. 3, S9). Similar models fit with a CI that was calculated using neighbors of all species produced less consistent responses across sites (not shown), suggesting differences in the competitive effects of conspecific vs. heterospecific neighbors. Thus our results suggest that managing competition in forests is likely to be important to promote resistance to mortality during drought (cf. [93], [94]). At the same time, it is important to note the apparent complexity involved, with species mix in addition to overall tree density needing consideration, as well as the potential role of stand structural characteristics beyond their influence on tree vigor (cf. [95]). Ultimately, further study is required to resolve the role of competition and tree density on mortality.

### Modeling Drought-associated Tree Mortality Using Growth-based Predictor Variables

Do simple metrics of tree productivity and carbon balance reflect the complex physiological, structural and life history aspects of individual trees that lead to their mortality during severe drought events? To our knowledge, our study represents the first to comprehensively assess and quantify growth-mortality relationships in the context of widespread drought-associated die-off, and it is one of only a few studies to look at the stability of growth-mortality relationships across space and time (but see [24]). Our models correctly classified ca. 70% of the trees across sites and drought events, which is comparable to or slightly below correct classification rates in other growth-mortality studies [29], [31]. Thus, there is promise in using relatively simple, growth-based empirical approaches for assessing drought-mortality risk, at least for certain species or functional groups, even when drought and associated insect activity causes rapid and widespread mortality.

However, we found variable growth-mortality relationships and thresholds between sites and drought events, and uneven model performance at the site level. Although individual sites did not exhibit opposite relationships between growth and mortality, as with background mortality in some European forests [96], the northern sites (TRP, WRK, BNM) were characterized by weaker associations between growth and mortality (Table 5; Fig. 2). This may represent a shift from mortality factors associated with chronic constraints on overall tree growth, to factors associated with shorter-term and/or exogenous factors. These factors include rapid hydraulic failure or carbon starvation in some more vigorous trees due to acute drought stress [40], [97], and/or the build-up of bark beetle populations that were able to overcome the resistance of trees with relatively higher growth rates [98]. Many bark beetles favor more vigorous trees with larger food stores, but they are only able to overcome the defenses of such trees at higher population densities [98]. The build-up of relatively large *Ips* populations may have been favored at some sites during the comparatively hot 2000s drought [49], as beetle development is accelerated by warm conditions [45].

Regardless of the underlying physiological causes, growth-mortality models calibrated on empirical data from the 1950s drought alone would have under-predicted mortality during the 2000s drought, thus underscoring the problems with projecting future mortality rates using empirical relationships established based on one drought event or experiment (see also [23]). If bark beetle attack is an underlying driver, integrating information on bark beetle dynamics with empirical indicators of tree physiological stress will be important for improving the predictive capacity of mortality models. Furthermore, our best growth-mortality models included longer-term, less simplistic growth metrics, suggesting that the time scales and/or cumulative processes considered by many current models should be extended (cf. [31]).

## Conclusions

Understanding the processes that underlie drought-related tree mortality is critical for anticipating future forest dynamics and associated feedbacks to the earth system, and for developing management plans that enhance the robustness and resilience of forests to climate change. Our study documented high levels of piñon mortality during both the 2000s and 1950s droughts, with almost ubiquitous evidence of bark beetle activity on dead trees. More synchronous mortality was observed at northern sites in New Mexico during the 2000s event. Dying trees generally had lower average growth rates and greater year-to-year growth variability than trees that survived, but early in their life, these differences were not evident. Instead, decades-long growth divergences between surviving and dying trees suggest that recent growth differences are related to the response and recovery of trees to previous severe droughts, at least at some sites. This pattern further suggests that a pool of trees that survived the early 2000s drought may now be particularly vulnerable during future droughts. These trees should be investigated in more detail to reveal the processes that influence their recovery [84].

We show that tree growth response to climate is an important predisposing factor underlying mortality during widespread, drought- and insect-related mortality events. In particular, our results suggest that tree response to wet/cool years, in addition to the response to drought years, may be an important aspect of vulnerability. The growth response of surviving trees during very wet/cool years in the decades preceding mortality events likely enhanced their carbon reserves, which was important for withstanding subsequent drought and insect attack. The competitive environment also influenced tree growth and the ability of trees to respond favorably to wet conditions, suggesting that controlling tree density is likely to enhance tree resistance to mortality during drought. However, conspecific vs. heterospecific competitive effects appear to be different, and should therefore be considered in detail.

The discriminatory ability of logistic growth-mortality functions underscores the potential of simple empirical approaches to represent mortality risk in models of vegetation dynamics, even in the context of widespread mortality events associated with drought and bark beetles. However, incorporating multiple and longer-term aspects of tree growth and life history is important for fully capturing mortality risk. Furthermore, shifting growth-mortality relationships across space and time point to the challenges associated with calibrating mortality algorithms. Although we cannot fully explain the weakening of growth-mortality relationships at the northern study sites during the 2000s drought, one consistent hypothesis is that bark beetle dynamics played a more important role in the recent die-off event, shifting the physiological basis for mortality and highlighting the need for further study of tree-insect dynamics to improve the prediction of tree mortality during drought.

## Supporting Information

Figure S1 **Study area locations within New Mexico, USA.** The distribution of *P. edulis* is shown in light gray.
(EPS)

Figure S2 **Relationships between tree diameter and radial growth as represented by different growth metrics.** Only live tree data is shown. A linear regression line with 95% confidence intervals is plotted over the raw data for relative basal area increments (RelBAI) (A), raw ring widths (RW) (B), basal area increments (BAI) (C), and ring width indices (RWI) (D). Trends are significant at the 95% level for (A) and (C), but not significant for (B) and (D). Growth is represented by an average of the most recent 3-years in each growth record.
(EPS)

Figure S3 **Size-class distributions of** ***Pinus edulis*** **that died and survived the 2000s drought.** Distributions are derived from measurements made in 7.5m neighborhood plots around each target tree. Bars in each histogram represent 2.5cm size classes, based on tree diameters at breast height (DBH). Data are pooled across all 2000s study sites in (A), and shown separately for TRP2000 (B), BNM2000 (C), WRK2000 (D) and SEV2000 (E). Size-class distributions for live and dead trees are significantly different overall (p<0.0001, Kolmogorov-Smirnov test), for TRP2000 (p = 0.0313), WRK2000 (p = 0.0006), and SEV2000 (0.0014). There were not enough living trees to test for differences at BNM2000.
(EPS)

Figure S4 **The relationship between tree density and mortality severity.** Each point represents the ratio of dead *Pinus edulis* (PIED) versus total tree density in 7.5m neighborhood-plots surrounding target trees at 2000s study sites ((A) TRP2000 (n = 60), (B) WRK2000 (n = 20) and (C) SEV2000 (n = 60)). A linear regression line is shown to provide a visual estimate of the relationship. Quasi-binomial regression was used to statistically assess the direction, magnitude and significance of depicted relationships. For TRP2000 (A), 0.00091x + 0.18240, p = 0.19. For WRK2000 (B), y = 0.00246x+0.06733, p = 0.17. For SEV2000 (C), y = –.00107x­– 0.22008, p = 0.021.
(EPS)

Figure S5 **Box and whisker plots of ring widths averaged over different time intervals.** Live tree growth is truncated at the outside ring date of the dead tree in the tree pair. Boxes drawn around time intervals on the x-axis denote significant differences between live and dead trees (p<0.05, Student’s t-test).
(EPS)

Figure S6 **Box and whisker plots of growth mean sensitivity averaged over different time intervals.** Live tree growth is truncated at the outside ring date of the dead tree in the tree pair. Boxes drawn around time intervals on the x-axis denote significant differences between live and dead trees (p<0.05, Student’s t-test).
(EPS)

Figure S7 **Box and whisker plots of tree growth trends averaged over different time intervals.** Live tree growth is truncated at the outside ring date of the dead tree in the tree pair. Boxes drawn around time intervals on the x-axis denote significant differences between live and dead trees (p<0.05, Student’s t-test).
(EPS)

Figure S8 **Box and whisker plots of the number of abrupt growth increases averaged over different time intervals.** Live tree growth is truncated at the outside ring date of the dead tree in the tree pair. Boxes drawn around time intervals on the x-axis denote significant differences between live and dead trees (p<0.05, Student’s t-test).
(EPS)

Figure S9 **Coefficients from models relating growth to climate, tree status and competition for trees that died and survived the 2000s drought.** Coefficients are from linear mixed-effects models relating growth (RW) to precipitation (PPT*_cool_*), vapor pressure deficit (VPD*_MJJ_*), competition (CI), and tree status (L/D). Coefficients were calculated separately for TRP2000 (A), WRK2000 (B), and SEV2000 (B). All predictor variables were converted to z-scores prior to modeling, allowing for a direct comparison of coefficients between models. Growth was modeled starting from five different dates leading up to the 2000s drought-mortality event (bar colors). The end of the modeled period varied depending on tree pair, with growth in surviving trees truncated at the outer year of growth in the corresponding dead tree.
(EPS)

Figure S10 **Coefficients from models relating growth to climate and tree status for trees that died and survived the 1950s drought.** Coefficients are from linear mixed-effects models relating growth (RW) to precipitation (PPT*_cool_*), vapor pressure deficit (VPD*_MJJ_*), and tree status (L/D). Coefficients were calculated separately for BNM1950 (A) and SEV50 (B). All predictor variables were converted to z-scores prior to modeling, allowing for a direct comparison of coefficients between models. Growth was modeled starting from five different dates leading up to the 1950s drought-mortality event (bar colors). The end of the modeled period varied depending on tree pair, with growth in surviving trees truncated at the outer year of growth in the corresponding dead tree.
(EPS)

Table S1 **Fine scale spatial patterning of mortality at 2000s sites.** Significant differences in tree density and basal area in neighborhood plots surrounding dead versus living target trees are in boldface type (p<0.05, Student’s t-test). PIED is *Pinus edulis*. JUMO is *Juniperus monosperma*.
(DOCX)

Table S2 **Bootstrapped estimates and confidence intervals for model terms in the best-ranked growth-mortality model.** The model formula is Tree Status ∼log(RW30) + Sens15 + AbruptIncreases10 + (1 + log(RW30) | Site), with validation statistics shown in Table 4. Variables include average growth (RW), mean sensitivity (Sens), and the number of abrupt growth increases (AbruptIncreases), with the number of years over which variables were averaged indicated after variable type. Bootstrapped estimates were generated by fitting models to 1000 samples drawn from the calibration data. The *Estimates* columns represent model coefficients for fixed effects and standard deviations for random effects.
(DOCX)

Text S1 **Justification for the choice of climate predictors in linear mixed-effects models.**
(DOCX)
