# Supplementary material for: Growth-Mortality Relationships in Piñon Pine (Pinus edulis) during Severe Droughts of the Past Century: Shifting Processes in Space and Time
Source: PLoS One. 2014 May 2;9(5):e92770. doi: 10.1371/journal.pone.0092770 (PMC4008371; doi:10.1371/journal.pone.0092770)
Supplement: Table S1 — Fine scale spatial patterning of mortality at 2000s sites. Significant differences in tree density and basal area in neighborhood plots surrounding dead versus living target trees are in boldface type (p<0.05, Student’s t-test). PIED is Pinus edulis. JUMO is Juniperus monosperma. (DOCX) [file pone.0092770.s011.docx]

|  | **TRP2000** | | | **WRK2000** | | | **SEV2000** | | |
| --- | --- | --- | --- | --- | --- | --- | --- | --- | --- |
|  | **Live** | **Dead** | **P-value** | **Live** | **Dead** | **P-value** | **Live** | **Dead** | **P-value** |
| **Density (trees ha^-1^)** |  |  |  |  |  |  |  |  |  |
| Total | 454.7 | 511.2 | 0.124 | 582.4 | 628.1 | 0.791 | 775.3 | 790.4 | 0.790 |
| PIED | **296.6** | **369.7** | **0.036** | 158.5 | 254.7 | 0.189 | 318.8 | 343.3 | 0.829 |
| JUMO | 101.5 | 84.9 | 0.437 | 384.8 | 345.2 | 0.761 | 399.9 | 390.5 | 0.661 |
| Dead PIED | **171.7** | **279.2** | **0.005** | 147.1 | 220.7 | 0.292 | 54.7 | 60.4 | 0.688 |
| Dead JUMO | 2.0 | 3.8 | 0.591 | 5.7 | 0.0 | 0.331 | 11.3 | 13.2 | 0.758 |
| **Basal Area (m^2^ ha^-1^)** |  |  |  |  |  |  |  |  |  |
| Total | 7.6 | 9.9 | 0.218 | 5.4 | 8.6 | 0.149 | 11.3 | 11.1 | 0.820 |
| PIED | 5.0 | 7.1 | 0.218 | 2.1 | 3.8 | 0.158 | 3.2 | 3.1 | 0.674 |
| JUMO | 2.6 | 2.8 | 0.982 | 3.0 | 4.7 | 0.211 | 8.2 | 8.0 | 0.654 |
| Dead PIED | **2.4** | **5.3** | **0.005** | 1.9 | 2.4 | 0.555 | 0.6 | 0.8 | 0.443 |
| Dead JUMO | 0.3 | 0.0 | 0.619 | 0.0 | 0.0 | 0.331 | 0.4 | 0.4 | 0.801 |
