# Supplementary material for: Growth-Mortality Relationships in Piñon Pine (Pinus edulis) during Severe Droughts of the Past Century: Shifting Processes in Space and Time
Source: PLoS One. 2014 May 2;9(5):e92770. doi: 10.1371/journal.pone.0092770 (PMC4008371; doi:10.1371/journal.pone.0092770)
Supplement: Table S2 — Bootstrapped estimates and confidence intervals for model terms in the best-ranked growth-mortality model. The model formula is Tree Status ∼log(RW30) + Sens15 + AbruptIncreases10 + (1 + log(RW30) | Site), with validation statistics shown in Table 4. Variables include average growth (RW), mean sensitivity (Sens), and the number of abrupt growth increases (AbruptIncreases), with the number of years over which variables were averaged indicated after variable type. Bootstrapped estimates were generated by fitting models to 1000 samples drawn from the calibration data. The Estimates columns represent model coefficients for fixed effects and standard deviations for random effects. (DOCX) [file pone.0092770.s012.docx]

|  | **Estimate** | **SE** | **Boot-strapped Estimate** | **Bootstrapped Range (95% CI)** |
| --- | --- | --- | --- | --- |
| **Fixed Effects** |  |  |  |  |
| (Intercept) | 4.437 | 1.171 | 4.851 | (3.231, 6.947) |
| log(RW30) | 2.799 | 0.893 | 3.089 | (2.037, 4.640) |
| Sens15 | -3.26 | 1.238 | -3.46 | (-6.046, -0.859) |
| AbruptIncreases10 | 0.208 | 0.07 | 0.224 | (0.078, 0.379) |
| **Random Effects** |  |  |  |  |
| Site: (Intercept) | 1.958 | - | 2.364 | (1.167, 4.128) |
| Site: log(RW30) (Intercept) | 1.62 | - | 1.955 | (0.941, 3.550) |
| Site: log(RW30) | 0 | - | 0.042 | (0.000, 0.415) |
