# Supplementary material for: Growth-Mortality Relationships in Piñon Pine (Pinus edulis) during Severe Droughts of the Past Century: Shifting Processes in Space and Time
Source: PLoS One. 2014 May 2;9(5):e92770. doi: 10.1371/journal.pone.0092770 (PMC4008371; doi:10.1371/journal.pone.0092770)
Supplement: Text S1 — Justification for the choice of climate predictors in linear mixed-effects models. (DOCX) [file pone.0092770.s013.docx]

**Justification for the choice of climate predictors in linear mixed-effects models**

Climate covariates in linear mixed-effects models were chosen based on initial comparisons between RWI mean-value chronologies and PRISM climate model output (4km resolution) [1]. We downloaded monthly (1898-2010) PRISM temperature, precipitation, and dew point data for each study site, and from these data calculated monthly precipitation minus potential evapotranspiration (P-PET) according to [2] and vapor pressure deficit (VPD) according to [3]. Monthly climate variables and RWI chronologies were entered as inputs to the program Seascorr, which utilizes correlation and bootstrapped estimations of correlation significance to assess the strength of relationships between growth chronologies and monthly, seasonal, and annual climatic variables [4]. RWI correlated most strongly with PRISM-derived precipitation and P-PET averaged over the nine-month period ending in May or June of the growth year, depending on site. May-July or May-August and previous September-November average temperature and VPD consistently resulted in the highest correlations between annual growth and temperature-related variables. These correlation windows are consistent with findings from other studies of conifer climate response in the region [3]. For ease of interpretation, we used 9-month cool season precipitation ending in May and May-July average VPD as the climatic predictors in mixed effects models presented here. Models using slightly different seasonal combinations of precipitation and VPD, as well average temperature and P-PET covariates, were also evaluated but are not shown because results were very similar.

**Literature Cited**

1. Daly C, Neilson RP, Phillips DL (1994) A Statistical-Topographic Model for Mapping Climatological Precipitation over Mountainous Terrain. Journal of Applied Meteorology 33: 140–158. doi:10.1175/1520-0450(1994)033<0140:ASTMFM>2.0.CO;2.

2. Hamon WR (1961) Estimating potential evapotranspiration. Proceedings of the American Society of Civil Engineers. Vol. 87. pp. 107–120.

3. Williams AP, Allen CD, Macalady AK, Griffin D, Woodhouse CA, et al. (2013) Temperature as a potent driver of regional forest drought stress and tree mortality. Nature Climate Change 3: 292–297. doi:10.1038/nclimate1693.

4. Meko DM, Touchan R, Anchukaitis KJ (2011) Seascorr: A MATLAB program for identifying the seasonal climate signal in an annual tree-ring time series. Computers & Geosciences 37: 1234–1241. doi:10.1016/j.cageo.2011.01.013.
